# Parkinson's disease inpatient mortality: Attention to sudden death

**DOI:** 10.1016/j.clinsp.2024.100366

**Published:** 2024-04-21

**Authors:** Tomás de la Rosa, Fúlvio Alexandre Scorza

**Affiliations:** a Neuroscience Department, Universidad de Cádiz, Cádiz, Spain; b Instituto de Investigación e Innovación Biomédica de Cádiz (INiBICA), Cádiz, Spain; c Centro de Investigación Biomédica en Red en Salud Mental (CIBERSAM), Instituto de Salud Carlos III, Madrid, Spain; d Neurology Department, Escola Paulista de Medicina Universidade Federal de São Paulo, São Paulo, SP, Brazil

Traditionally, Parkinson's Disease (PD) has been depicted in research as primarily characterized by its motor symptoms.^1^ The progression of the disease frequently leads to a range of debilitating manifestations, encompassing diminished mobility, cognitive decline resulting in dementia, and autonomic dysfunction. Moreover, individuals affected by PD exhibit heightened mortality rates when compared with demographically matched cohorts,^2^ a trend that has been notably exacerbated in recent years. This is something concerning given the anticipated doubling of PD prevalence by 2050, attributable to global population aging, with projections estimating a population of 2.1 billion individuals affected.^3^ Thus, PD is not only a degenerative, chronic and progressive disease, but also a deadly one. With this in mind, the authors approach the article by Phillips and colleagues^4^ with considerable interest, as it illuminates the rising occurrence of mortality and its associated risk factors among hospitalized PD patients. Their data, gathered from around half a million patients, illustrates a steady rise in mortality rates among PD inpatients from 2002 to 2016 in the US, compared to individuals hospitalized for other medical conditions. A logistic regression model was conducted, revealing elevated odds of mortality among male, white, and older patients. However, these predictors demonstrated significance only in earlier periods of the data, implying evolving trends concerning the influence of these sociodemographic variables. But there also were significantly greater odds of inpatient mortality in those patients with greater illness severity and mortality risk, measured at admission. As anticipated, the highest odds were observed in the extreme subclasses of both disease severity (OR = 6.45) and mortality risk (OR = 61.9), prompting the authors to suggest that this information should be utilized to guide patients and their families in determining the preferred location of death. Nevertheless, significantly increased mortality rates were also noted in the moderate disease severity (OR = 1.51) and mortality risk (OR = 3.13) subclasses. The authors acknowledge limitations in their study, notably that the NIS database, from which the data was extracted, did not include information on PD severity or cause of death. This limitation restricts the understanding of inpatient mortality occurrences among individuals who were not initially classified with major or extreme severity or mortality risk.

Common causes of death in PD overlap with age-matched populations, and despite extensive research on disease progression and symptom management, mortality in PD research is sometimes underexplored in the literature. Cardiovascular and respiratory issues are the leading causes of mortality among PD patients, and it has been recently acknowledged a growing occurrence of sudden and unexpected deaths in individuals diagnosed with PD, referred to as “sudden unexpected death in Parkinson's disease” (SUDPAR).^5^^,^^6^ SUDPAR is characterized as an unexpected death in PD patients without any definitive cause established through autopsy studies.^7^ It represents a classification of death observed among individuals with PD, rather than being a manifestation of the disorder itself, posing challenges for investigation in translational studies due to its rarity and the limitations of postmortem examinations. Some risk factors have been identified as potential contributors to this phenomenon, including age at disease onset, gender, or polypharmacy.^6^ Although the mechanisms remain unclear, some evidence points towards a role of autonomic dysfunctions and cardiac abnormalities.^8^

The data provided by Phillips et al.^4^ shows how inpatient mortality is associated with disease severity and mortality risk, something that is in line with previous epidemiological data that associated PD motor severity impairment, dementia or age with increased mortality.^9^ SUDPAR as a cause of death might be associated with disease progression.^5^^,^^6^ However, there is a lack of data concerning its incidence in hospitalized patients and its correlation with variables specific to such settings, as examined in the study. It remains unclear if there is a linear correlation between PD severity, as assessed by the Hoehn & Yahr scales or other parkinsonism impairment scores, and the All Patient Refined Diagnosis Related Groups (APR-DRG) methodology utilized in the study. Patients classified within the moderate subclass of this scale, exhibiting heightened mortality rates compared to controls, may manifest increased vulnerability to SUDPAR. As given by its definition as a sudden and unexpected death, this phenomenon could potentially impact individuals who were considered stable at the time of admission. The gap between APR-DRG scores and the individual risk of SUDPAR may partially account for the mortality observed in the study. The emphasis on SUDPAR among inpatients stems from the understanding that hospitalized patients represent a group with heightened potential for both, prevention and thorough study and comprehension of the phenomenon.

The authors find it crucial to better address this challenge posed to PD research and care. It is necessary to gather a comprehensive clinical history, including an assessment of familiar medical history, focusing on other cardiovascular risk factors such as smoking or obesity, that could trigger this event. Moreover, a thorough physical examination is needed, and routine cardiovascular screenings are employed when deemed necessary by specialists. This requires a close collaboration in the clinical setting between cardiologists and neurologists, to prevent these deaths among hospitalized PD patients. Finally, to gain a deeper understanding of the incidence, risk factors, and mechanisms associated with SUDPAR, clinical and preclinical multidisciplinary studies should be undertaken, employing approaches that successfully unveil the links between APR-DRG scores, PD phenotypes and progression, and SUDPAR events.

## Authors’ contributions

Both TR and FS contributed equally to the conceptualization, writing, and reviewing of the present manuscript.

## Funding

The present studies are supported by the following grants: FAPESP (Fundação de Amparo à Pesquisa do Estado de São Paulo); CNPq (Conselho Nacional de Desenvolvimento Científico e Tecnológico), Coordenação de Aperfeiçoamento de Pessoal de Nível Superior (CAPES) and ‘Juan de la Cierva’ grant financed by (MICIU/AEI/10.13039/501100011033) and by European Social Fund (FSE+).

## Declaration of competing interest

The authors declare that the research was conducted in the absence of any commercial or financial relationships that could be construed as a potential conflict of interest.
